# Gabapentinium picrate

**DOI:** 10.1107/S1600536809008952

**Published:** 2009-03-19

**Authors:** Hongqi Li, H. S. Yathirajan, L. Mallesha, K. N. Mohana, B. Narayana

**Affiliations:** a Key Laboratory of Science and Technology of Eco-Textiles, Ministry of Education, College of Chemistry, Chemical Engineering and Biotechnology, Donghua University, Shanghai 201620, People’s Republic of China; b Department of Studies in Chemistry, University of Mysore, Manasagangotri, Mysore 570 006, India; c Department of Studies in Chemistry, Mangalore University, Mangalagangotri 574 199, India

## Abstract

The title compound {systematic name: [1-(carboxy­meth­yl)cyclo­hexyl]methanaminium 2,4,6-trinitro­phenolate}, C_9_H_18_NO_2_^+^·C_6_H_2_N_3_O_7_^−^, was synthesized from picric acid and gabapentin. The crystal packing is stabilized by intra­molecular N—H⋯O=N and N—H⋯O—Ph hydrogen bonds. An O—H⋯O inter­action is also present.

## Related literature

For background, see: Bryans & Wustrow (1999 ▶). For related structures, see: Ibers (2001 ▶); Swamy *et al.* (2007 ▶) and references cited therein.
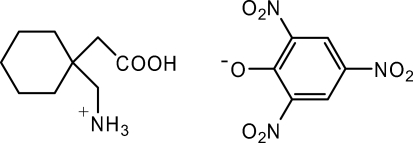


## Experimental

### 

#### Crystal data

C_9_H_18_NO_2_^+^·C_6_H_2_N_3_O_7_^−^
*M* *_r_* = 400.35
Monoclinic, 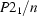

*a* = 11.576 (2) Å
*b* = 7.7312 (16) Å
*c* = 19.973 (4) Å
β = 91.425 (2)°
*V* = 1787.0 (6) Å^3^
*Z* = 4
Mo *K*α radiation
μ = 0.12 mm^−1^
*T* = 296 K
0.25 × 0.25 × 0.20 mm

#### Data collection

Bruker SMART CCD area-detector diffractometer
Absorption correction: multi-scan (*SADABS*; Sheldrick, 2004 ▶) *T*_min_ = 0.970, *T*_max_ = 0.976
8899 measured reflections
3150 independent reflections
2408 reflections with *I* > 2σ(*I*)
*R*_int_ = 0.034

#### Refinement

*R*[*F* ^2^ > 2σ(*F* ^2^)] = 0.062
*wR*(*F* ^2^) = 0.182
*S* = 1.07
3150 reflections
256 parameters
H-atom parameters constrained
Δρ_max_ = 1.06 e Å^−3^
Δρ_min_ = −0.55 e Å^−3^

### 

Data collection: *SMART* (Bruker, 2001 ▶); cell refinement: *SAINT* (Bruker, 2001 ▶); data reduction: *SAINT*; program(s) used to solve structure: *SHELXS97* (Sheldrick, 2008 ▶); program(s) used to refine structure: *SHELXL97* (Sheldrick, 2008 ▶); molecular graphics: *SHELXTL* (Sheldrick, 2008 ▶); software used to prepare material for publication: *SHELXTL*.

## Supplementary Material

Crystal structure: contains datablocks global, I. DOI: 10.1107/S1600536809008952/cs2105sup1.cif

Structure factors: contains datablocks I. DOI: 10.1107/S1600536809008952/cs2105Isup2.hkl

Additional supplementary materials:  crystallographic information; 3D view; checkCIF report

## Figures and Tables

**Table 1:** Hydrogen-bond geometry (Å, °)

*D*—H⋯*A*	*D*—H	H⋯*A*	*D*⋯*A*	*D*—H⋯*A*
O9—H9⋯O8^i^	0.82	1.86	2.672 (3)	174
N4—H4*C*⋯O4^ii^	0.89	2.32	2.957 (4)	128
N4—H4*C*⋯O3^ii^	0.89	2.06	2.862 (3)	149
N4—H4*B*⋯O2^iii^	0.89	2.41	2.894 (3)	114
N4—H4*B*⋯O4^i^	0.89	2.23	3.063 (3)	155
N4—H4*A*⋯O3^iii^	0.89	2.21	3.081 (3)	166

Symmetry codes: (i) 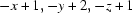; (ii) 
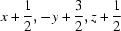
; (iii) 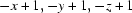.

**Fig. 1.**
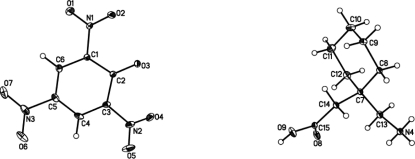
A view of the asymmetric unit of the title compound. Displacement ellipsoids are drawn at the 30% probability level and H atoms are shown as small spheres of arbitrary radii.

**Fig. 2.**
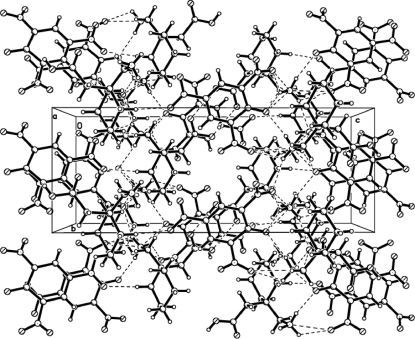
Packing in the crystal structure of the title compound, viewed along the a axis

## supplementary crystallographic information

### Comment

Gabapentin, (1-(aminomethyl) cyclohexane acetic acid; Neurontin), is a novel anticonvulsant agent and has therapeutically beneficial effects against chronic pain states and anxiety (Bryans & Wustrow, 1999). Gabapentin is a zwitterion in the solid state (Ibers, 2001). On the other hand, picric acid forms salts or charge-transfer complexes with many organic compounds and we have reported crystal structures of a number of picrate complexes with organic compounds of pharmaceutical importance *viz*. desipraminium picrate (Swamy *et al.*, 2007). The present paper reports the crystal structure of the title compound, (1-(carboxymethyl)cyclohexyl)methanaminium 2,4,6-trinitrophenolate.

### Experimental

The title compound was synthesized by mixing solutions of picric acid (4.59 g, 0.01 mol) in 20 ml of distilled water and gabapentin (1.72 g, 0.01 mol) in 20 ml of distilled water and the resulting solution was stirred well for 10 min. A yellow precipitate of gabapentinium picrate was formed almost instantaneously after stirring. The so formed yellow complex was filtered off, washed with distilled water and dried *in vacuo* over anhydrous calcium chloride. The compound was purified by successive recrystallization from methanol (yield 92%). Single crystals for X-ray studies were grown by slow evaporation of a methanol solution. Analysis (%) found (calculated) for C_15_H_20_N_4_O_9_: C: 44.68 (45.00); H: 5.11 (5.04); N: 13.73 (13.99)%.

### Refinement

All H atoms were placed at calculated positions and refined using a riding model approximation, with C—H = 0.93–0.97 Å and with *U*_iso_(H) = 1.2*U*_eq_(C).

### Crystal data

**Table:**

C_9_H_18_NO_2_^+^·C_6_H_2_N_3_O_7_^−^	*F*(000) = 840
*M**_r_* = 400.35	*D*_x_ = 1.488 Mg m^−^^3^
Monoclinic, *P*2_1_/*n*	Melting point = 431–434 K
Hall symbol: -P 2yn	Mo *K*α radiation, λ = 0.71073 Å
*a* = 11.576 (2) Å	Cell parameters from 2666 reflections
*b* = 7.7312 (16) Å	θ = 2.8–25.1°
*c* = 19.973 (4) Å	µ = 0.12 mm^−^^1^
β = 91.425 (2)°	*T* = 296 K
*V* = 1787.0 (6) Å^3^	Block, yellow
*Z* = 4	0.25 × 0.25 × 0.20 mm

### Data collection

**Table:**

Bruker SMART CCD area-detector diffractometer	3150 independent reflections
Radiation source: fine-focus sealed tube	2408 reflections with *I* > 2σ(*I*)
graphite	*R*_int_ = 0.034
φ and ω scans	θ_max_ = 25.1°, θ_min_ = 2.8°
Absorption correction: multi-scan (*SADABS*; Sheldrick, 2004)	*h* = −12→13
*T*_min_ = 0.970, *T*_max_ = 0.976	*k* = −7→9
8899 measured reflections	*l* = −23→23

### Refinement

**Table:**

Refinement on *F*^2^	Secondary atom site location: difference Fourier map
Least-squares matrix: full	Hydrogen site location: inferred from neighbouring sites
*R*[*F*^2^ > 2σ(*F*^2^)] = 0.062	H-atom parameters constrained
*wR*(*F*^2^) = 0.182	*w* = 1/[σ^2^(*F*_o_^2^) + (0.083*P*)^2^ + 1.5949*P*] where *P* = (*F*_o_^2^ + 2*F*_c_^2^)/3
*S* = 1.07	(Δ/σ)_max_ < 0.001
3150 reflections	Δρ_max_ = 1.06 e Å^−^^3^
256 parameters	Δρ_min_ = −0.55 e Å^−^^3^
0 restraints	Extinction correction: *SHELXL97* (Sheldrick, 2008), Fc^*^=kFc[1+0.001xFc^2^λ^3^/sin(2θ)]^-1/4^
Primary atom site location: structure-invariant direct methods	Extinction coefficient: 0.075 (5)

### Fractional atomic coordinates and isotropic or equivalent isotropic displacement parameters (Å^2^)

**Table:**

	*x*	*y*	*z*	*U*_iso_*/*U*_eq_	
C1	0.2919 (2)	0.3970 (4)	0.05392 (13)	0.0375 (6)	
C2	0.3067 (2)	0.5162 (3)	0.10930 (13)	0.0362 (6)	
C3	0.3639 (2)	0.6728 (3)	0.08832 (16)	0.0432 (7)	
C4	0.3949 (2)	0.7066 (4)	0.02348 (17)	0.0499 (8)	
H4	0.4323	0.8093	0.0131	0.060*	
C5	0.3701 (2)	0.5869 (4)	−0.02563 (15)	0.0466 (8)	
C6	0.3199 (2)	0.4315 (4)	−0.01060 (14)	0.0433 (7)	
H6	0.3052	0.3503	−0.0441	0.052*	
C7	0.4810 (2)	0.6530 (3)	0.67642 (12)	0.0328 (6)	
C8	0.5192 (2)	0.4847 (3)	0.71209 (13)	0.0372 (6)	
H8A	0.6030	0.4811	0.7144	0.045*	
H8B	0.4920	0.4871	0.7576	0.045*	
C9	0.4752 (3)	0.3205 (4)	0.67810 (17)	0.0521 (8)	
H9A	0.5112	0.3084	0.6350	0.063*	
H9B	0.4972	0.2213	0.7053	0.063*	
C10	0.3458 (3)	0.3223 (5)	0.6679 (2)	0.0727 (11)	
H10A	0.3093	0.3214	0.7111	0.087*	
H10B	0.3216	0.2192	0.6437	0.087*	
C11	0.3074 (3)	0.4818 (5)	0.6289 (2)	0.0664 (10)	
H11A	0.2237	0.4833	0.6247	0.080*	
H11B	0.3381	0.4771	0.5842	0.080*	
C12	0.3490 (3)	0.6467 (4)	0.66370 (16)	0.0502 (8)	
H12A	0.3109	0.6569	0.7062	0.060*	
H12B	0.3261	0.7453	0.6364	0.060*	
C13	0.5043 (3)	0.8069 (4)	0.72276 (14)	0.0429 (7)	
H13A	0.4866	0.9122	0.6982	0.051*	
H13B	0.4517	0.8003	0.7597	0.051*	
C14	0.5472 (2)	0.6665 (3)	0.61064 (12)	0.0354 (6)	
H14A	0.6290	0.6669	0.6222	0.042*	
H14B	0.5319	0.5615	0.5853	0.042*	
C15	0.5244 (2)	0.8160 (3)	0.56474 (13)	0.0365 (6)	
N1	0.2450 (3)	0.2259 (3)	0.06681 (13)	0.0530 (7)	
N2	0.3944 (3)	0.8022 (3)	0.13862 (17)	0.0582 (8)	
N3	0.4040 (2)	0.6182 (5)	−0.09448 (17)	0.0633 (9)	
N4	0.6251 (2)	0.8199 (3)	0.75081 (12)	0.0490 (7)	
H4A	0.6443	0.7210	0.7710	0.074*	
H4B	0.6292	0.9060	0.7803	0.074*	
H4C	0.6734	0.8404	0.7178	0.074*	
O1	0.1815 (4)	0.1631 (5)	0.02309 (16)	0.1380 (18)	
O2	0.2579 (2)	0.1585 (3)	0.11974 (12)	0.0681 (8)	
O3	0.2754 (2)	0.4861 (3)	0.16754 (10)	0.0492 (6)	
O4	0.3311 (2)	0.8242 (3)	0.18624 (13)	0.0638 (7)	
O5	0.4804 (3)	0.8876 (4)	0.1315 (2)	0.1066 (12)	
O6	0.4584 (2)	0.7510 (5)	−0.10551 (16)	0.0911 (10)	
O7	0.3773 (3)	0.5105 (5)	−0.13704 (14)	0.0893 (10)	
O8	0.4688 (2)	0.9447 (3)	0.57786 (10)	0.0609 (7)	
O9	0.5759 (2)	0.7959 (3)	0.50733 (11)	0.0616 (7)	
H9	0.5576	0.8754	0.4820	0.092*	

### Atomic displacement parameters (Å^2^)

**Table:**

	*U*^11^	*U*^22^	*U*^33^	*U*^12^	*U*^13^	*U*^23^
C1	0.0436 (15)	0.0344 (14)	0.0344 (14)	−0.0022 (12)	−0.0046 (11)	0.0041 (11)
C2	0.0400 (14)	0.0318 (14)	0.0364 (15)	0.0020 (11)	−0.0054 (11)	0.0019 (11)
C3	0.0420 (15)	0.0297 (14)	0.0575 (18)	−0.0008 (12)	−0.0075 (13)	0.0030 (13)
C4	0.0384 (16)	0.0382 (16)	0.073 (2)	0.0036 (13)	0.0079 (14)	0.0239 (16)
C5	0.0394 (15)	0.0549 (19)	0.0457 (16)	0.0112 (14)	0.0059 (12)	0.0156 (15)
C6	0.0428 (16)	0.0514 (18)	0.0356 (15)	0.0031 (13)	−0.0026 (12)	0.0021 (13)
C7	0.0415 (14)	0.0297 (13)	0.0271 (13)	0.0035 (11)	0.0006 (10)	0.0015 (10)
C8	0.0490 (16)	0.0323 (14)	0.0301 (14)	−0.0001 (12)	−0.0014 (11)	0.0044 (11)
C9	0.073 (2)	0.0313 (16)	0.0520 (18)	−0.0097 (15)	−0.0040 (15)	0.0041 (13)
C10	0.076 (3)	0.058 (2)	0.084 (3)	−0.031 (2)	0.002 (2)	0.004 (2)
C11	0.0460 (19)	0.078 (3)	0.074 (2)	−0.0127 (18)	−0.0084 (16)	−0.001 (2)
C12	0.0416 (16)	0.059 (2)	0.0505 (18)	0.0065 (14)	0.0054 (13)	0.0072 (15)
C13	0.0624 (19)	0.0337 (15)	0.0325 (14)	0.0073 (13)	0.0012 (13)	−0.0009 (11)
C14	0.0466 (15)	0.0311 (14)	0.0285 (13)	0.0016 (11)	0.0016 (11)	0.0015 (10)
C15	0.0480 (16)	0.0346 (15)	0.0268 (13)	−0.0010 (12)	−0.0009 (11)	0.0005 (11)
N1	0.0781 (19)	0.0435 (15)	0.0369 (14)	−0.0202 (13)	−0.0054 (12)	−0.0054 (12)
N2	0.0603 (17)	0.0320 (14)	0.082 (2)	−0.0054 (13)	−0.0139 (16)	−0.0025 (14)
N3	0.0510 (16)	0.078 (2)	0.0612 (19)	0.0195 (16)	0.0141 (14)	0.0320 (18)
N4	0.0808 (19)	0.0321 (13)	0.0337 (13)	−0.0104 (12)	−0.0085 (12)	−0.0038 (10)
O1	0.240 (5)	0.111 (3)	0.0613 (19)	−0.105 (3)	−0.031 (2)	0.0042 (18)
O2	0.0992 (19)	0.0492 (14)	0.0547 (15)	−0.0240 (13)	−0.0203 (13)	0.0170 (11)
O3	0.0769 (15)	0.0367 (11)	0.0340 (11)	−0.0009 (10)	0.0009 (10)	0.0000 (8)
O4	0.0849 (18)	0.0401 (13)	0.0655 (16)	0.0009 (12)	−0.0174 (14)	−0.0124 (11)
O5	0.089 (2)	0.072 (2)	0.159 (3)	−0.0438 (18)	0.005 (2)	−0.029 (2)
O6	0.0774 (19)	0.101 (2)	0.096 (2)	0.0071 (17)	0.0278 (16)	0.0562 (19)
O7	0.106 (2)	0.116 (3)	0.0471 (15)	0.016 (2)	0.0227 (15)	0.0115 (17)
O8	0.1037 (19)	0.0422 (13)	0.0375 (12)	0.0242 (13)	0.0137 (11)	0.0090 (10)
O9	0.0925 (18)	0.0544 (14)	0.0389 (12)	0.0224 (13)	0.0198 (11)	0.0148 (10)

### Geometric parameters (Å, °)

**Table:**

C1—C6	1.363 (4)	C10—H10B	0.9700
C1—C2	1.447 (4)	C11—C12	1.524 (5)
C1—N1	1.455 (4)	C11—H11A	0.9700
C2—O3	1.249 (3)	C11—H11B	0.9700
C2—C3	1.447 (4)	C12—H12A	0.9700
C3—C4	1.377 (4)	C12—H12B	0.9700
C3—N2	1.455 (4)	C13—N4	1.497 (4)
C4—C5	1.374 (5)	C13—H13A	0.9700
C4—H4	0.9300	C13—H13B	0.9700
C5—C6	1.371 (4)	C14—C15	1.495 (4)
C5—N3	1.460 (4)	C14—H14A	0.9700
C6—H6	0.9300	C14—H14B	0.9700
C7—C13	1.527 (4)	C15—O8	1.217 (3)
C7—C14	1.541 (3)	C15—O9	1.314 (3)
C7—C8	1.543 (3)	N1—O2	1.185 (3)
C7—C12	1.544 (4)	N1—O1	1.228 (4)
C8—C9	1.522 (4)	N2—O5	1.205 (4)
C8—H8A	0.9700	N2—O4	1.227 (4)
C8—H8B	0.9700	N3—O7	1.225 (4)
C9—C10	1.506 (5)	N3—O6	1.227 (4)
C9—H9A	0.9700	N4—H4A	0.8900
C9—H9B	0.9700	N4—H4B	0.8900
C10—C11	1.520 (5)	N4—H4C	0.8900
C10—H10A	0.9700	O9—H9	0.8200
			
C6—C1—C2	124.9 (3)	C10—C11—H11A	109.4
C6—C1—N1	116.3 (3)	C12—C11—H11A	109.4
C2—C1—N1	118.8 (2)	C10—C11—H11B	109.4
O3—C2—C1	124.2 (2)	C12—C11—H11B	109.4
O3—C2—C3	124.8 (3)	H11A—C11—H11B	108.0
C1—C2—C3	111.0 (2)	C11—C12—C7	113.7 (3)
C4—C3—C2	124.2 (3)	C11—C12—H12A	108.8
C4—C3—N2	117.0 (3)	C7—C12—H12A	108.8
C2—C3—N2	118.7 (3)	C11—C12—H12B	108.8
C5—C4—C3	119.3 (3)	C7—C12—H12B	108.8
C5—C4—H4	120.4	H12A—C12—H12B	107.7
C3—C4—H4	120.4	N4—C13—C7	115.4 (2)
C6—C5—C4	121.1 (3)	N4—C13—H13A	108.4
C6—C5—N3	118.5 (3)	C7—C13—H13A	108.4
C4—C5—N3	120.3 (3)	N4—C13—H13B	108.4
C1—C6—C5	119.4 (3)	C7—C13—H13B	108.4
C1—C6—H6	120.3	H13A—C13—H13B	107.5
C5—C6—H6	120.3	C15—C14—C7	119.4 (2)
C13—C7—C14	112.4 (2)	C15—C14—H14A	107.5
C13—C7—C8	109.4 (2)	C7—C14—H14A	107.5
C14—C7—C8	107.9 (2)	C15—C14—H14B	107.5
C13—C7—C12	106.5 (2)	C7—C14—H14B	107.5
C14—C7—C12	111.9 (2)	H14A—C14—H14B	107.0
C8—C7—C12	108.7 (2)	O8—C15—O9	122.6 (2)
C9—C8—C7	114.1 (2)	O8—C15—C14	125.9 (2)
C9—C8—H8A	108.7	O9—C15—C14	111.5 (2)
C7—C8—H8A	108.7	O2—N1—O1	121.3 (3)
C9—C8—H8B	108.7	O2—N1—C1	121.2 (2)
C7—C8—H8B	108.7	O1—N1—C1	117.0 (3)
H8A—C8—H8B	107.6	O5—N2—O4	121.8 (3)
C10—C9—C8	111.9 (3)	O5—N2—C3	118.9 (3)
C10—C9—H9A	109.2	O4—N2—C3	119.4 (3)
C8—C9—H9A	109.2	O7—N3—O6	124.5 (3)
C10—C9—H9B	109.2	O7—N3—C5	118.1 (3)
C8—C9—H9B	109.2	O6—N3—C5	117.3 (4)
H9A—C9—H9B	107.9	C13—N4—H4A	109.5
C9—C10—C11	110.7 (3)	C13—N4—H4B	109.5
C9—C10—H10A	109.5	H4A—N4—H4B	109.5
C11—C10—H10A	109.5	C13—N4—H4C	109.5
C9—C10—H10B	109.5	H4A—N4—H4C	109.5
C11—C10—H10B	109.5	H4B—N4—H4C	109.5
H10A—C10—H10B	108.1	C15—O9—H9	109.5
C10—C11—C12	111.0 (3)		

### Hydrogen-bond geometry (Å, °)

**Table:**

*D*—H···*A*	*D*—H	H···*A*	*D*···*A*	*D*—H···*A*
O9—H9···O8^i^	0.82	1.86	2.672 (3)	174
N4—H4C···O4^ii^	0.89	2.32	2.957 (4)	128
N4—H4C···O3^ii^	0.89	2.06	2.862 (3)	149
N4—H4B···O2^iii^	0.89	2.41	2.894 (3)	114
N4—H4B···O4^i^	0.89	2.23	3.063 (3)	155
N4—H4A···O3^iii^	0.89	2.21	3.081 (3)	166

Symmetry codes: (i) −*x*+1, −*y*+2, −*z*+1; (ii) *x*+1/2, −*y*+3/2, *z*+1/2; (iii) −*x*+1, −*y*+1, −*z*+1.
